# Affective Barriers to Accessing Professionalized Intimate Partner Violence Services Among LGBTQ People in Australia

**DOI:** 10.1177/10778012251323237

**Published:** 2025-03-17

**Authors:** Gene Lim, Stephanie Lusby, Marina Carman, Adam Bourne

**Affiliations:** 1 110434 Australian Research Centre in Sex, Health, and Society, La Trobe University, Melbourne, Victoria, Australia; 2 Respect Victoria, Melbourne, Victoria, Australia; 3 Safe and Equal, Victoria, Melbourne, Victoria, Australia

**Keywords:** LGBTQ, intimate partner violence, help-seeking, IPV services, Australia

## Abstract

LGBTQ victim-survivors of intimate partner violence (IPV) encounter numerous obstacles to accessing professionalized support, including internal factors that engender reluctance to engage in professionalized services. This gap in knowledge constitutes a limiting factor to the uptake of these services, even when significant effort has been made to accommodate these individuals. Semi-structured life history interviews were conducted with (*N* = 30) LGBTQ victim-survivors aged 19–79 years with recent (<2 years) and/or ongoing IPV experiences. These factors functionally curtailed access to appropriate support, even when available. The implementation of inclusive services must be attentive to and address affective barriers encountered by LGBTQ victim-survivors

## Introduction

Intimate partner violence (IPV) within the lesbian, gay, bisexual, trans, and gender diverse and queer (LGBTQ)^1^ population constitutes a pressing public health concern that has historically been absent from both scholarship and policy settings (Edwards et al., 2020; Lay et al., 2018; Scheer & Poteat, 2021). IPV is commonly understood as comprising discrete behavioral patterns that are collectively calibrated to coerce, control, or incite fear; these may even include criminal assault or property damage (Rathus, 2013; Stark & Hester, 2019). IPV is linked to negative mental health outcomes, including depression and PTSD (Woulfe & Goodman, 2020), reduced financial and housing security (McNair et al., 2018), jeopardization of employment or social support (e.g., as may result from the non-consensual disclosure of one's sexual and/or gender identity) (Gray et al., 2023; Workman & Dune, 2019), threats related to one's visa or migration status (Cisneros & Bracho, 2020; Murray et al., 2019; Vaughan et al., 2016), as well as damage to property, harm to dependants, and physical injury (Australian Institute of Health and Welfare, 2019; Messinger, 2020; Rollè et al., 2018; Yerke & DeFeo, 2016).

Recent evidence suggests that IPV occurs within LGBTQ populations at comparable or higher rates relative to the general population (Brown & Herman, 2015; Victorian Agency for Health Information, 2020; Walters et al., 2013). LGBTQ victim-survivors additionally encounter unique dimensions of IPV, and their experiences of violence are often informed by and enmeshed with extant forms of marginalization and oppression. This is further exacerbated by considerable obstacles that impede LGBTQ victim-survivors’ access to professionalized services such as police, courts, specialist family violence response providers, and refugees (Victorian Agency for Health Information, 2020). Safe, inclusive, and responsive services that are optimized to meet the unique needs of LGBTQ individuals are indispensable to victim-survivor recovery (Carman et al., 2020; McKay et al., 2019).

However, the provision of such services can be further complicated by the fact that sexual and/or gender minority status rarely constitutes the sole form of oppression or marginalization within most LGBTQ individuals’ lives. While unified by common experiences of heterosexism and/or cissexism, the needs of LGBTQ populations are otherwise characterized by significant heterogeneity. This arises from a matrix of sociodemographic factors like race, geographic location, financial and/or housing stability, experience of disability, immigration status, and past experiences of violence or discrimination (Campo, 2015; Seymour, 2019). These social variations shape individual LGBTQ victim-survivors’ recognition of their experiences as IPV (Lusby et al., 2022) and furthermore inform the accessibility and perceived availability of support services.

This article seeks to explore the affective barriers to help-seeking from professionalized services faced by LGBTQ people experiencing IPV and examines ways that these barriers may mutually inform and reinforce one another. Drawing on life history interviews with 30 LGBTQ victim-survivors with recent (<2 years) experience of IPV, this study addresses the ongoing need for scholarship and practice to better understand the barriers to service support faced by LGBTQ victim-survivors. The present discussion defines “professionalized” services as a mainstream specialist family and domestic violence and sexual violence (FDSV) services; police and courts; LGBTIQ community organizations that provide counseling and other support to people experiencing IPV and sexual violence; and national telephone helplines for people experiencing IPV and sexual violence.

### Family and Domestic Violence: The Public Narrative

Both the insidious nature and accretionary impact of IPV, as well as its presentation within LGBTQ families and relationships, are often omitted from the “public story” around violence and abuse (Donovan & Hester, 2011). Historically, domestic violence has predominantly been framed as physical violence perpetrated by cisgendered, heterosexual men against cisgendered, heterosexual women within the context of an intimate relationship (MacDonald, 1998; Serra, 2012). Over the past decade, there has been a more comprehensive move toward acknowledging patterns of coercive control (Donovan & Barnes, 2020c; Stark & Hester, 2019) and nonphysical abuse tactics in domestic policy and law (Rathus, 2013).

Despite this, the abiding public narrative of IPV is one which both frames it as a quintessentially hetero-gendered phenomenon and which emphasizes physical violence as an identifying characteristic. These preconceptions often obfuscate LGBTQ victim-survivors’ recognition of their experiences as abuse, given that they are typically unable to identify with these narratives, or recognize themselves within them (Donovan & Barnes, 2020a; Messinger, 2011). While this intuitively constitutes a barrier to help-seeking behaviors among LGBTQ victim-survivors, evidence suggests that these narratives also inflect service providers’ awareness of LGBTQ victim-survivors’ needs. Through this narrative lens, LGBTQ victim-survivors are often positioned as “niche” and as ancillary to those of cisgender women. This, in turn, underpins the inadequate resourcing that most mainstream IPV services receive to support LGBTQ persons experiencing violence (Lim et al., 2023). This, in turn, undermines LGBTQ victim-survivors’ trust in service capability and diminishes the perceived availability of LGBTQ-inclusive services (Lim et al., 2023).

### LGBTQ-Specific Contours of Intimate Partner Violence

From a socio-ecological perspective, scholars have identified several common impediments to help-seeking among LGBTQ victim-survivors across a variety of jurisdictions (Santoniccolo et al., 2023). These chiefly relate to LGBTQ victim-survivors’ ability to recognize their experiences as violence. In many settings, low levels of knowledge about violence in LGBTQ relationships and of nonphysical forms of abuse (Donovan & Hester, 2011; Freeland et al., 2018; Harden et al., 2022) are often concomitant with the normalization of violence within same-sex relationships (Harden et al., 2022). LGBTQ victim-survivors may therefore experience “negative support” (Santoniccolo et al., 2023), wherein disclosures about one's experiences of IPV are met with minimization, aspersion, and downplayed by others (Harden et al., 2022). Other scholars have drawn attention to how adherence to rigid gender stereotypes informs LGBTQ individuals’ experiences of violence; for instance, causing IPV between men to be seen as a normative expression of masculinity (Oliffe et al., 2014), or contributing to its minimization when perpetrated by women (Bates et al., 2019).

Negative societal attitudes toward nonheterosexual and trans and gender-diverse identities and individuals also facilitate unique forms of violence, chief among these being “identity abuse” (Woulfe & Goodman, 2020). Persons using abuse may attempt to weaponize these attitudes and direct them against a victim-survivor through the nonconsensual disclosure of the victim-survivor's sexual and/or gender minority identity (Harden et al., 2022). This can jeopardize a victim-survivor's employment opportunities, as well as their family or social relationships in ways that may be intended to increase their reliance on the person using abuse (Harden et al., 2022). Another set of tactics similarly conceptualized as “identity abuse” within the literature relates to the delegitimization or denigration of a victim-survivor's sexual and/or gender identity and is calibrated to leverage a target's internalized homo/bi/transphobia against them (Woulfe & Goodman, 2020).

These forms of abuse are salient even within the context of LGBTQ+ relationships. In mixed-orientation relationships, for instance, where one partner is gay or lesbian and the other multigender-attracted (e.g., bisexual, or pansexual), abusive tactics may revolve around the invalidation of the latter's sexual identity (Bermea et al., 2018; Head, 2020). Woulfe and Goodman (2020) point out that identity abuse frequently leverages extant oppressions to effect control. In the above instance, the invalidation of bi+ identities often arises from the heterosexist notion that monosexuality (e.g., attraction to a single gender) is the default configuration of human sexuality (McCann, 2022). Likewise, “identity abuse” leveraged against a trans partner may reference foundationally cissexist beliefs that prescribe a narrow range of gender identities as legitimately “trans,” and/or more blatantly transphobic beliefs pertaining to the illegitimacy of trans identities all together (Anderson, 2020; Marrow, 2023). Hence, even as LGBTQ individuals are marginalized and disadvantaged by the oppressive structures of heterosexism and cissexism, they are often able to weaponize these self-same structures against their partners.

### Affective Barriers to Help-Seeking among LGBTQ Victim-Survivors

Several common material barriers to help-seeking are experienced by LGBTQ victim-survivors across otherwise distinct jurisdictions. This includes a lack of professionalized support services that are adequately equipped to render support to LGBTQ victim-survivors (Lusby et al., 2024; Lusby et al., 2022), a combination of both real and anticipated discrimination from service providers (Lim et al., 2023), as well as a raft of sexual identity-agnostic factors that impact service accessibility for most victim-survivors (Donovan & Barnes, 2020a, 2020b, 2020c; Scheer et al., 2020). Comparatively less well-understood are the affective dimensions of the help-seeking process—in particular, how certain affective processes and experiences may impact victim-survivor access to support structures. Present scholarship offers well-articulated insights into how (presumed cisgender, heterosexual) female victim-survivors’ help-seeking behaviors are shaped by the affective dimensions of their abuse (McCleary-Sills et al., 2016; Voth-Schrag et al., 2021). In comparison, a similarly comprehensive exploration of the role that shame and stigma play in informing help-seeking LGBTQ victim-survivors is presently nascent.

Stigma surrounding the experience of victimization, as well as feelings of shame and embarrassment are well-established as common characteristics of affective experiences of abuse among both heterosexual (Heron et al., 2022) and LGBTQ individuals (Anderson & Overby, 2020). These feelings of shame and negative potentiate outcomes for victim-survivors and may even increase the likelihood of future IPV (Thaggard & Montayre, 2019). Chiefly, shame-proneness is often linked to poorer mental health outcomes and may predict higher levels of posttraumatic stress disorder (PTSD) in victim-survivors, while simultaneously underpinning negative expectations of support from others (Aakvaag et al., 2016). Shame can also complicate both help-seeking as well as recovery—causing victim-survivors to delay help-seeking and to avoid disclosing their experiences of abuse (Thaggard & Montayre, 2019). The negative affective consequences experienced by victim-survivors are therefore not simply an unpleasant subjective state but are actively detrimental to victim-survivors’ socioemotional health and well-being.

Present evidence suggests that LGBTQ victim-survivors are particularly impacted in this regard, given that their feelings of embarrassment and shame surrounding their experiences of abuse may be compounded by the perception that their experiences confirm negative stereotypes about same-sex relationships. This “stereotype threat,” often experienced as a desire to defy stereotypical perceptions of LGBTQ relationships as inherently dysfunctional or unhealthy further, may contribute to a reluctance to name one's experiences as abuse (Donovan & Barnes, 2020c). Particularly in Australia, where LGBTQ relationships and unions were very recently the subject of public scrutiny (Perales & Todd, 2018), LGBTQ individuals may perceive their relationships as being the subject of undue notice and may feel pressured to uphold the image of LGBTQ relationships as healthy and functional (Lim et al., 2023). Feelings of shame and stigma are recognized within the literature as directly contributing to IPV within LGBTQ relationships (Harden et al., 2022), as well as a deterrent to LGBTQ victim-survivors’ help-seeking behaviors (Rollè et al., 2020). At the time of writing, however, there is little available evidence examining the other affective barriers that might impede LGBTQ victim-survivors from seeking help.

### Understanding LGBTQ Victim-Survivors’ Engagement With Support Services

Extant scholarship demonstrates that LGBTQ victim-survivors often do not view “professionalized” services as viable sources of support and may instead perceive them as either potential sources of discrimination and/or as ill-equipped to support the needs of LGBTQ persons (Lusby et al., 2022). Consequently, LGBTQ individuals may perceive little recourse than to seek out assistance from various sources, often in a piecemeal fashion—reaching out to family, friends, or community (Sylaska & Edwards, 2014) or existing clinical supports such as general practitioners or mental health professionals (Santoniccolo et al., 2021).

Other barriers relate to the availability of inclusive IPV services, as well as the uptake of said services where available. Scholars have previously noted consistently low rates of service uptake among LGBTQ victim-survivor populations across several jurisdictions (Messinger, 2014). Likewise, professionalized support may be unevenly available across LGBTQ subpopulations. For instance, while crisis accommodation or support groups may be accessible to lesbian, bisexual, and even transgender women within some jurisdictions, these same services may exclude gay, bisexual, and transgender men (Messinger, 2014, p.150). Neither are LGBTQ individuals’ experiences within these services homogenous; for instance, extant literature suggests that transgender women may be exposed to transmisogynistic discrimination from women's services (Messinger et al., 2022), whereas LGBTQ individuals who experience needs relating to their cultural or racial identities may experience considerable difficulty accessing services where their needs can be holistically met (Lim et al., 2023).

These factors are often collectively interpreted as an indication that LGBTQ victim-survivors largely eschew the use of “mainstream” professionalized services or limit their engagement exclusively to services population-specific services (Barnes & Donovan, 2018; Head, 2020). However, it is increasingly evident that this characterization of LGBTQ victim-survivors’ help-seeking behaviors is an oversimplification and that LGBTQ victim-survivors may engage with “mainstream” services even where there is a chance that they may experience discriminatory attitudes from service providers (Lim et al., 2023). LGBTQ victim-survivors’ utilization of “mainstream” professionalized services may occur due to the immediacy of one's needs, a lack of nonprofessionalized support, an unwillingness to access nonprofessionalized support, and/or a sheer lack of population-specific alternatives (Donovan & Barnes, 2020; Gray et al. 2020; Lay, Leonard & Parsons, 2018; O’Halloran, 2015).

## The Current Study

In summary, LGBTQ victim-survivors in Australia encounter unique barriers to accessing safe, inclusive, and appropriate IPV services. While many of these barriers relate to material or structural factors and are well-explored within the IPV literature, present understandings of the affective barriers to service engagement and uptake among LGBTQ victim-survivors within Australia are nebulously understood by comparison. To address this gap in the literature, the current study drew upon 30 qualitative interviews with LGBTQ victim-survivors to investigate how affective factors shape these individuals’ help-seeking practices, intentions, and expectations.

## Research Methods and Design

### Methodology

A qualitative methodology was used in the present study. Qualitative studies are relatively scarce within IPV-related research (Barnes & Donovan, 2018), with the preponderance of quantitative methodologies reflecting hegemonic conceptualizations of IPV as a public health issue (Donovan & Hester, 2011). However, the nuances of individual, lived experience are difficult to properly capture using quantitative methodologies (Bergin, 2018). This is particularly true for topics such as IPV, which are linked to deeply entrenched structures of marginality and disadvantage (Garcia, López & Vélez, 2018).

### Recruitment

Participants were recruited via an email invitation to a database of LGBTIQ+ people who had previously identified their interest in being contacted about new research projects. Prospective participants were provided with a link to a screening survey that ensured those contacted for interview met the study inclusion criteria and collected demographic information about their gender, sexuality, age, ethnicity, state of residence, and geographical residence. The following conditions determined participation eligibility; participants had to: (i) identify as LGBTQ victim-survivors, (ii) be above 18 years of age, (iii) reside in Australia, and (iv) have experienced family, domestic or sexual violence in the preceding 2 years. A total of 3,412 individuals were invited to participate in the screening survey, which recorded 204 responses. Purposive sampling was used to obtain diverse representation across all sociodemographic categories in the study, and we arrived at a finalized sample comprising (*N* = 30) participants.

### Data Collection

Interviews were structured as a narrative or life history—centered upon a recent experience of family, domestic and/or sexual violence, and experiences of help-seeking. Participants were asked about recent experiences of abuse related to intimate partner violence (IPV), sexual violence experienced in the context of casual or dating encounters, violence perpetrated by a housemate, and violence within a participant's family of origin, either as an adult or a minor. They were also asked about their experiences access support, whether these attempts resulted in improvements in their safety or well-being, and the reasons why people chose not to approach professionalized services for help. Diversity within the sample meant that participants were able to speak about their experience of disability; navigating care of children and custody arrangements in the context of family violence; and interactions with institutions and services, including police, courts, national helplines, LGBTQ community organizations, mainstream FDSV services, GPs, psychologists and counseling services, migration agencies, hospitals, and social workers.

A key strength of the chosen approach is that participants can share multiple experiences and communicate the complexity of their stories in ways that made sense to them, while also centering questions around the barriers and enablers to accessing services. This “grounds these stories of personal experience in their wider social and historical context and pays attention to social relations of power” (Bathmaker & Harnett, 2010, p. 2). Participants are also afforded greater agency and control in framing their own narrative, focusing on the events and timelines that are important to them (Riessman, 2008), facilitating a generative meaning-making process (Golden, 2020). This facilitated participant introspection about the different interpretations or meanings of the recounted events.

Interviews were conducted remotely due to COVID-19 public health orders in operation at the time, and to facilitate representation from across Australia, including regional and rural areas as well as urban centers. Twenty-nine interviews were conducted by the second author, a cisgender heterosexual woman of Anglo-Celtic descent. One interview was conducted by the last author, a cisgender gay man of Anglo-Celtic descent. The research team drew on safety protocols used by IPV service providers providing remote counseling or assistance to inform our approach to minimizing risk to participant safety (see Lim et al., 2023). This included emphasizing the importance of their being in a space where they would not be overheard and could speak freely, ensuring that they were feeling mentally and emotionally well enough to talk about their experiences of IPV, and giving participants authority over how the interview proceeded in the event of emotional distress.

Audio recordings were transcribed into text through a paid transcription service and uploaded onto NVivo12.0 for manual coding by both the first author—a cisgender bisexual man of Singaporean Chinese descent—and the second author. A preliminary coding framework was developed using the first and last authors’ field notes and reflections on the collected data, as well as findings from a previous phase of the project that involved interviews with professionalized IPV service providers. This was informed by the principles of thematic analysis, as articulated by Braun & Clarke (2006). The first and second authors both engaged in line-by-line coding using this preliminary framework, which was gradually expanded in both scope and complexity. Following a first round of coding, the second and last authors convened to discuss expansions to the coding framework, ensure adequate code application, and resolve discrepancies in code assignments. The finalized coding framework contained 6 parent codes and 30 subcodes, combining both semantic and latent themes.

### Participant Characteristics

Participants were aged between 19 and 79 years old. A relatively even distribution of transgender (*N* = 14) and cisgender (*N* = 15) participants was observed. Participants identifying as queer (*N* = 12) were the best-represented group within our sample, while roughly equal numbers of gay (*N* = 9), lesbian (*N* = 7), and bisexual (*N* = 6) participants were noted. Most participants were of Anglo-Celtic descent (*N* = 21), and we noted a small contingent of individuals were of Pan-African (*N* = 2) and Ashkenazi Jewish descent (*N* = 2), respectively. Only a single Aboriginal/Torres Strait Islander participant participated in these interviews.

### Ethics

Institutional ethics approval was granted for this study by the La Trobe University Human Research Ethics Committee (HREC). All participants are referred to hereafter using pseudonyms in the interest of maintaining confidentiality.

## Findings

Our analyses yielded four distinct themes and suggest that affective factors broadly impact LGBTQ victim-survivors’ willingness to engage with professionalized services by: (i) detracting from their ability to recognize and name their experiences as IPV, and (ii) affecting victim-survivors’ perceptions of service availability and/or suitability. Furthermore, these affective factors were underpinned by: (iii) deficits of trust in professionalised institutions and services, as well as (iv) unique relational consequences perceived to arise from disclosing one's experiences of abuse. While these factors are not categorically unique to sexual and gender minority persons, our finding provides convincing evidence to suggest that their manifestation within the context of LGBTQ IPV is underlined by a raft of historic, systemic, and institutional considerations unique to LGBTQ individuals. Below, we outline how these experiences are further modulated by a matrix of sociodemographic, biographical, and individual factors in ways specific to the circumstances of each participant.

### Recognizing and Naming Experiences of IPV

A common theme within participant narratives (*n = 23*) pertained to how naming and recognizing IPV within one's experiences was undermined by implicit beliefs about IPV that aligned with its lay definitions—as detailed in previous sections. This abiding image of IPV seemed to impact even those participants with prior knowledge of, and/or exposure to LGBTQ-specific manifestations of IPV. In particular, the emphasis on physical violence as a legitimizing component of IPV frequently prevented participants from recognizing intimate partners’ actions as IPV. However, even when physical violence featured prominently within patterns of abuse, the omission of LGBTQ perspective from these lay narratives nevertheless meant that some participants did not reliably name their experiences as IPV (*n *= 9).

For many participants (*n = 20*), the lack of an overarching, normative frame upon which a coherent understanding of their experiences of abuse as IPV could be scaffolded clearly shaped the affective contours of participants’ experiences of abuse, as well as their experiences of help-seeking. In some instances (*n = 11*), the lack of recognition underpinned the notion that one's experiences were atypical and defied normative expectations of IPV palpably contributed to participants’ sense that they were “shunned and expelled from human connectedness” (Walker, 2011, p. 452).

One participant, Janelle (Anglo-Celtic, Cisgender Woman, Lesbian), spoke of the tensions between her initial understanding of abuse within LGBTQ relationships and her coworkers’ concern at her worsening job performance and visible bruising upon her person. For Janelle, the reactions of others around her were a nebulous warning “reflecting back to me that perhaps something wasn’t totally right” within her relationship. Yet her inability to recognize and name her experiences as abuse meant that instead of enlightening her to the nature of her situation, this instead caused her to feel “incredibly isolated and invisible and un-legitimized.”

In other instances, this inability to recognize IPV within the context of same-sex relationships colluded with other factors to impede the recognition of one's experiences as abuse, and thus delayed help-seeking. One participant, Justine, reflected on numerous examples of abusive, or otherwise problematic behaviors exhibited which ultimately eluded recognition as such. For instance, she recalled several occasions where her partner had attempted to strike her but remarked that she never considered this a credible threat to her personal safety, due to her ex-partner's relatively diminutive physical stature. It was only when she discovered an ornamental, but still-functional firearm among her ex-partner's possessions that she understood the imminent danger she was in. In the immediate aftermath, however, Justine described a conversation with a friend where she dismissed the possibility that she was experiencing domestic violence:

I’d just for the first time called the police about a gun, where I used to live with the ex. And I handed in this firearm to the police and my partner was away that day and then it was afterwards I rang a dear friend of mine, because I was quite shaken. And she goes, “Justine, this is all domestic violence.” And I’m going, “No. No. Bullshit…. I work in mental health. She hasn't - no, I'm still here. I'm not battered and bruised.” –Justine, Anglo-Celtic, Cisgender Woman, Lesbian.

Justine's accounts were further notable in that they demonstrate how implicit beliefs about IPV—in this case, of its synonymity with physical abuse, collide with personal factors—specifically, Justine's confidence in her ability to identify IPV as a mental health professional—to obfuscate the recognition of abusive behavioral patterns within a victim-survivor's intimate relationship.

For other participants such as Justine, the shame attached to their victimhood was further compounded by feelings of self-blame. Many recounted feeling that they ought to have recognized the tell-tale signs of abuse, with many participants describing self-recriminatory thoughts that they had somehow “allowed” themselves to be victimized. Another participant, Goldie, had worked for much of her career in roles focused on addressing gender-based violence, including IPV, and “gender inequality.” At several points in her interview, she articulated her discomfort with what she felt to be an inconsonance between her professional identity and the abuse endured during her previous relationship, stating:

I am an educated, articulate woman who's worked in this field, and I think [that was] the shame that I felt through that period, to not be able to talk to anyone about it, because I should’ve known better – Goldie, Anglo-Celtic, Cisgender Woman, Lesbian

This misalignment between her professional identity and her experiences of victimization was a source of shame to Goldie, which in turn contributed to a reluctance to name her experiences as IPV—despite a rational awareness that IPV could happen to anybody.

The sense of shame attached to one's experiences of victimization was not unique to individuals who held professionalized knowledge about IPV and was noted as a common affective response to the feelings of disempowerment that resulted from participants’ experiences of abuse. This was observably the case for Gerald, a cisgender gay man in his 70s who spoke of how elements of elder and financial abuse gradually crept into his relationship with a younger ex-partner. Gerald spoke proudly of their considerable professional success as a corporate executive earlier in his life. Consequently, his lack of control over the situation, as well as the difficulty he encountered in attempting to leave his partner became a source of frustration for Gerald, who felt that it reflected poorly on his competence. He stated:

I thought that I could handle all this. I’ve done some very important jobs in the past, I had lots of responsibilities, there was no reason why I shouldn’t handle this properly, and I’d be able to deal with a character like this, no problem at all. Then I heard this program [about emotional abuse], and I thought: ‘No…hang on, Perhaps I can’t. Perhaps I’m vulnerable.’ – Gerald, Anglo-Celtic, Cisgender Man, Gay

Participant narratives such as these illustrate the pernicious impact of narratives that frame victim-survivors as “weak or passive” (Donovan & Hester, 2010, p. 282). Additionally, these narratives ascribe a quintessentially “feminine” quality to experiences of victimization and victim-survivors themselves. This initially causes participants like Gerald to perceive these narratives as being of little relevance to his experiences—preventing him from naming his experiences as IPV, while also underpinning the shame he experienced when he ultimately recognizes his experiences as abuse. Participants like Gerald, Justine, and Goldie, whose professional identities are starkly contrasted against these stereotypic perceptions of victim-survivors were therefore likely to disidentify with these understandings of victim-survivor-hood. Where this prefaces a failure of recognition for both Justine and Goldie, individuals like Gerald are instead motivated to preserve their sense of self-efficacy by pursuing a resolution that eschews any external support. In both cases, these narratives visibly contribute to victim-survivors’ underestimation of their own needs in relation to professionalized support.

Yet another affective barrier to help-seeking pertained to victim-survivors’ diffidence in naming their experiences of abuse as IPV. Here, participants appraised their individual experiences against highly prescriptive narratives of IPV that almost invariably framed physical violence and bodily injury as hallmarks of IPV. Particularly for individuals whose experiences of abuse involved threats of violence, but not literal physical harm, their experiences existed nebulously within this paradigm. Even as they recounted the violence they had suffered, some participants remained tentative about characterizing their experiences as abuse (*n = 7*). Jia Hao, a gay Asian man living in a regional town with an older white partner was one such individual. Jia Hao spoke at length about his partner's violent outbursts which were wholly directed toward inanimate household objects, as well as his threats of eviction and overall emotional volatility. While the participant was intellectually able to recognize these behaviors as IPV, he was visibly conflicted about naming his experiences as such. Even after self-nominating to participate in our study, he voiced his uncertainty by saying:

You can have a hard line to say you have to physically abuse someone, and then there's people that sort of say control and coercion and the verbal … you know, there's a whole spectrum, so it's very hard to, sort of, know. I guess I’m happy to talk about [my experiences], but there's always that fear that maybe I wasn’t really in a domestic violence relationship and I’m wasting your time – Jia Hao, Han Chinese, Cisgender Man, Gay.

Past scholarship attests to how experiences of abuse can erode victim-survivors’ sense of self-worth and confidence in their own instincts and judgment (Ko & Park, 2020; Hing et al., 2021). However, Jia Hao's uncertainty was furthermore noted to be informed by previous experiences of racialization specific to the intersection of sexual and racial identity that he occupied.

In describing his peer networks—many of which were dominated by white gay men—Jia Hao noted that there was a tendency for him to assume the stereotypical role of “the Asian toy boy, [where] you get spoken to rather than have any ideas you know, you just put up with it. You become this servant type person.” Therefore, the habituation of racially minoritized victim-survivors to asymmetrical racial dynamics in their social relationships with white persons (e.g., such as where the words and opinions of the latter are arbitrarily afforded more weight and credence) understandably undermines these victim-survivors’ sense of self-efficacy. This is perhaps doubly so in circumstances such as Jia Hao's, whose experiences of violence occur at the hands of a white partner.

### Perceptions of Service Availability and/or Suitability

While an indispensable facilitator of help-seeking processes among victim-survivors, recognition of one's experiences as IPV, in and of itself, was seldom the sole obstacle to accessing support. Oftentimes, participants’ willingness to seek support was nevertheless offset by a perceived lack of available or suitable services (*n = *19). This was particularly the case for cisgender men, as well as trans and gender-diverse participants, who typically perceived “mainstream” services as primarily intended for and optimized to cisgender women. Lance, a pansexual man living in an urban center stated:

I think that you know some of the mainstream DV organisations that you might go to, even if it's as simple as calling [national sexual violence and IPV hotline] or some of the crisis lines, they're really for battered women, they're not for lesbian women, they're not for gay men, they're not for transgender people, and they probably wouldn’t have a clue…I can remember not reaching out to mainstream DV organisations because of that, and also the embarrassment and humiliation which comes with being male as well.

– Lance, Anglo-Celtic & African, Cisgender Man, Pansexual.

Lance's account illustrates the interplays between individual and societal factors in shaping victim-survivors’ affective orientations toward their experiences of abuse and of subsequent help-seeking. This quote also highlights how perceived disjoints between one's experiences of victimization and conventional masculine gender norms compound upon perceptions of service unavailability or unsuitability to constrain viable support options for some victim-survivors.

Despite residing in a large capital city, Lance further noted a lack of LGBTQ-specific IPV services. He commented that most LGBTQ victim-survivors were forced to turn to community-led LGBTQ organizations which, at the time, lacked any dedicated organizational capacity or expertise for supporting victim-survivors. Hence, even though Lance recounts being subject to severe—and on one occasion, potentially grievous—bodily harm by his ex-partner, nowhere in his interview does he recount any kind of engagement with professionalized IPV support services. Lance instead describes intermittently accessing a bricolage of health services, which was supplemented by nonprofessionalized sources of support from various friends and coworkers.

These perceptions of service unsuitability were also shared by several transgender women (*n = *3) but stemmed largely from the gender-essentialist paradigms that were perceived to guide service delivery or mainstream IPV services. Zelda, a transgender woman whose occupation brought her in regular contact with FDSV service providers, expressed her disquiet toward the implicitly binary gendered-practice frameworks used by many “mainstream” services. Trans and gender-diverse individuals, who sit nebulously within these dichotomous conceptualizations of gender, likewise voiced concern that they would be viewed through the lens of their assigned sex. For transgender women specifically, this is compounded by the equivalence of maleness with IPV perpetration within those paradigms. The confluence of these factors led many transgender women to judge that their experiences of abuse were liable to be disbelieved—particularly if the person using abuse was a cisgender woman—as was the case for Zelda.

Zelda was particularly adamant that these binary-gendered practice frameworks were definitionally incompatible with an LGBTQ-inclusive service outlook. She was insistent that even if concessions were explicitly made for transgender women by these services, these frameworks are foundationally incompatible with the provision of trans-inclusive care:

…even if you’re like, ‘oh no, I accept trans women or women,’ … if you’re like all men are bad, then it's like well you know, while you were living as a man you were a bad man. And so, there's just that kind of implicit transphobia in that, I just don’t think you can get around it – Zelda, Anglo-Celtic, Tran Woman, Queer.

While Zelda's views are not informed by firsthand experience, they were nevertheless informed inferences that referenced: (i) past experiences of being excluded by IPV services while she still identified as a man, (ii) second-hand accounts from trans peers about their experiences with said services to the effect described above, and (iii) firsthand experiences with inconsistent approaches to trans-inclusivity from services in a professional capacity. Examples like these demonstrate how affective barriers can often serve a distinct, protective function that enables victim-survivors to avoid discrimination from service providers during a window of special vulnerability.

In Zelda's case, these affective barriers must be contextualized against the infiltration of trans-exclusionary ideologies into some “mainstream” service organizations under the guise of radical feminism (Lim et al., 2023). Through this ideological lens, transgender women are reduced to their presumed gender at birth, and so viewed as beneficiaries of patriarchal power structures who leverage their male privilege to gain unfettered access to women-only spaces. Zelda explains:

They think they’re punching up at men but they’re actually punching down at queer people by making it uncomfortable for us to access any of those services… unless they were incredibly explicit about it, I would assume that I would be excluded as a trans woman

– Zelda, Anglo-Celtic, Tran Woman, Queer.

The presumed motives of transgender women are therefore to impose upon these spaces as a means of preying on vulnerable women (Harden et al., 2022). Zelda states that these beliefs often embolden, and even morally compel its adherents to deny transgender women much-needed support.

### Deficits of Trust

“Deficits of trust” (Donovan & Hester, 2011) were a thematically related affective barrier that pertained to participants’ diffidence in specific institutions and agencies’ ability to engage with them in a productive and without prejudice. Many affective barriers stemming from these deficits of trust arose at the conjunction of negative personal experiences with specific professionalized services, and extant histories of oppressive conduct in relation to minoritized groups by specific institutions. Hence, far from being unfounded or irrational, these deficits of trust should be understood as legitimate and intuitive responses that reflect the ongoing treatment minoritized groups experience from these institutions.

### Policing Services

Participants largely voiced a lack of confidence in the ability of law enforcement officers to appreciate the unique dynamics inherent to nonheterosexual relationships (*n = *17)—and were perceived as being liable to misidentify the perpetrator of abuse (*n = *8). For both cisgender and transgender women in same-sex relationships, this fear was rooted in the equivocation of femininity with victimhood—which participants anticipated would result in the minimization of their experience of being abused by another woman. Conversely, both cis and trans men expressed concern that the association between maleness and IPV perpetration within these same narratives might cause them to be misidentified as the individuals wielding violence.

This was succinctly articulated by Lance, who was reluctant to involve the police, despite being seriously injured by his boyfriend on multiple occasions:

On the subject of being male, feeling like you know I might be mistaken as the perpetrator […] And then you know there was that added level of humiliation around I’m male you know, I’m not a battered woman, I should as far as masculine standards go have, I should have the capability to fight back, and I didn’t –Lance, Anglo-Celtic & African, Cisgender Man, Pansexual.

Both here and elsewhere, Lance demonstrates a keen awareness of the history of prejudiced policing against marginalized communities and individuals. Lance's perspectives were likely also shaped by his engagement in sex work and history of drug addiction—particularly since his partner's abusive behaviors specifically targeted these characteristics. Hence, while Lance's previous interactions with the police are recounted as an even mix of both positive and negative encounters, he nevertheless comes to prejudge this as an unviable avenue of support.

### Mental Health Support

Similar deficits of trust had less dramatic, but nonetheless enduring effects on victim-survivors’ preferences in support services. Tony, a brotherboy^2^ in his mid-20s, spoke about his experiences with mental health professionals as an adolescent. He described these initial encounters as nonconsensual and occurring in the context of a custody dispute between his parents. Tony drew direct throughlines from these experiences to his present-day preferences, noting:

I used counselling services on the phone, just because of where I lived and also – I don’t really trust psychologists like ongoing, so I struggle to build the trust […] I had an experience with psychologists and counsellors where I wasn’t – I wasn’t consenting to the service as a teenager […] it was like trauma counselling. And it just went like too far asking all these questions, it was for child safety related reasons

–Tony, Aboriginal, Transmasculine/Brother Boy, Bisexual/Queer.

Here, Tony details a defining encounter with mental health professionals in a face-to-face setting, wherein an indelicate approach to probing his experiences of parental abuse appears to foreground his discomfort with engaging in such services. Doubts about whether mental health professionals would treat gender-diverse individuals like himself with dignity and care were further layered over his preexisting reservations. Elsewhere, Tony's quotes demonstrated a rational awareness that many IPV support services had made significant strides in implementing inclusive service provision for trans and gender-diverse individuals. Nevertheless, this was insufficient to overcome the entrenched distrust toward such services. As a result, Tony describes predominantly accessing crisis support services, instead of forming long-term therapeutic alliances.

Another expression of these deficits is related to participants’ skeptical attitudes toward service workers’ commitments to upholding patient–practitioner confidentiality (*n = *4). Troublingly, some concerns relating to this theme were articulated by individuals who had some form of professional or social involvement with individual IPV service workers. One particularly striking instance of this was recounted by Helena, who had observed several lapses in patient–practitioner confidentiality among acquaintances who worked within the sector: commitment to upholding patient–practitioner confidentiality. Troublingly, some concerns relating to this theme were articulated by individuals who had some form of professional or social involvement with individual IPV service workers. One particularly striking instance of this was recounted by Helena, who had observed several lapses in patient–practitioner confidentiality among acquaintances who worked within the sector:

I don’t trust anonymity and I believe that there are perpetrators amongst service providers, and I’ve heard people sitting around, people who are you know domestic violence support workers and counsellors, just bitching and speaking violently about [victim-survivors] – Helena, Anglo/Other European, Non-Binary, Pansexual.

Accounts like Helena's highlight the vigilance generally observed among victim-survivors whilst navigating help-seeking and further emphasize how these affective barriers are fundamentally erected in the service of minimizing further emotional harm.

### Embedded and Entangled

Another category of affective barriers pertained to service organizations that were embedded or enmeshed with pre-existing community structures. Community outreach and integration are indispensable components of service accessibility for LGBTQ victim-survivors (Lusby et al., 2022). However, participants (*n = *8) expressed concern that these close community affiliations had the potential to create uncomfortable or embarrassing situations, citing such concerns to qualify their aversion toward community-led LGBTQ-specific services. Such concerns were generally unrelated to the standards of confidentiality or professionalism in these services but focused instead on the chance for incidental encounters with other members of what were oftentimes compact and highly interconnected communities. Participants’ feelings of shame and embarrassment toward their experiences of victimization diminished their willingness to access these services. For example, Amelia, asocial worker living in the suburban fringes of a major city stated:

There wasn’t [any] help for me through LGBTI community organisations because I work in the LGBTI role, and I run a queer network…and I’ve worked in identified LGBTI roles in [city] for a long time, so there just isn’t that option. Someone at one of the main services, they are really close with my ex…so yeah, they're just not a safe person to speak to about this stuff

– Amelia, Anglo-Celtic, Non-Binary (AFAB), Bisexual/Queer.

Amelia's ex-partner was similarly embedded within the same social networks alongside many of the frontline service workers within her jurisdiction. This introduced an additional affective barrier to Amelia's help-seeking journey. Most professionalized services likely observe ethical protocols that safeguard against such scenarios (e.g., by ensuring that victim-survivors are not assigned to service workers with whom they have a preexisting social relationship). Nevertheless, Amelia demonstrably regards the potential for her professional and personal lives to co-mingle as an insurmountable deterrent against accessing such services.

Similar concerns likewise complicated the help-seeking intentions and behaviors of individuals like Sam, whose experiences of abuse occurred while they were residing in a small, nonurban community. While technically able to access a local population-specific service as well as a handful of mainstream professionalized services, Sam nevertheless evaluated neither as tenable avenues of support. Firstly, previous employment in the former, and a romantic past with a staff member currently employed by said service were cited as factors that ruled out engaging with this population-specific service for Sam. They state:

I didn’t get any extra support that I could have, I also could have reached out to someone like [service]… but I used to work for [service]… I’ve slept with one of them in [that] office, it's a pretty small office, it's a small community, everyone knows each other…Again it's embarrassing, it's airing dirty laundry and stuff like that – Sam, Anglo-Celtic, Gender-Diverse, Queer.

Concurrently, Sam explains their aversion to utilizing the local mainstream services, feeling that word about their experiences of abuse would quickly spread throughout the highly interconnected, socially conservative community they resided in:

I did have the opportunity to then go to DV services but it's so heavily gendered and so straight and we’re in a country town, it was over in [regional centre] anyway which is an hour away … there's confidentiality and privacy concerns, you know, in the country, in a way that doesn’t exist in the city. I didn’t want to see somebody and have them see me down the shops later. I know that they’re a professional and they can separate those things out but it's still there because you know you feel embarrassed.

– Sam, Anglo-Celtic, Gender-Diverse, Queer.

Participants who, like Sam, resided in non-urban localities similarly experienced a raft of unique relational factors that constrained the perceived viability of local services as avenues of support. Within these contexts, the boundaries between one's personal and public lives are often perceived as highly porous, such that the risk of incidental sexual identity disclosure was viewed as a perennial concern. As LGBTQ individuals’ acceptance within these communities can often hinge on their image as “good” and “ordinary” rural persons (Kayzak, 2012), being outed within these contexts may expose these individuals to heightened levels of homophobic discrimination (Marlin et al., 2022).

## Discussion

The present study identified four overarching motifs that broadly characterized the affective barriers to help-seeking for LGBTQ victim-survivors. Namely, these were: (i) an inability to recognize and name one's experiences as IPV, (ii) perceptions of service availability and viability, (iii) deficits of trust in specific service institutions, and (iv) perceived relational consequences tied to disclosing one's experiences of abuse. In all the accounts cited above, these affective barriers are informed by a raft of relational, structural, historic, and systemic factors that coalesce in a diversity of permutations to inflate participants’ perceptions of the risks associated with help-seeking and service engagement.

In naming the subjective nature of these deterrents to service engagement, our intention is not to imply that the help-seeking behaviors demonstrated by participants were purely the result of feared or anticipated discrimination. Rather, the exploration of these affective deterrents is intended to highlight a hitherto largely underexplored impediment to service access among LGBTQ victim-survivors. Indeed, both participants’ life histories and marginal identities suggest that the ample caution exercised by these participants is both prudent and appropriate. For many participants, feelings of shame and embarrassment and their impact on decisions regarding help-seeking were indicative of the defensive posture from which many victim-survivors sought support. These patterns of help-seeking behaviors spoke to an underlying impetus to minimize further encounters with trauma for participants, whilst preserving preexisting social relationships.

Hence, participants’ affective responses to help-seeking fulfill an important, protective function. However, as our previous research demonstrates, these same affective responses can also impede victim-survivors’ access to genuinely inclusive and safe care and can significantly delay the return on service organizations’ investments in building LGBTQ-inclusive capabilities (see: Lusby et al., 2022). These affective barriers therefore constrain victim-survivors’ timely access to safe and inclusive services and may obstruct service access even in the technical absence of issues relating to service availability or accessibility. While lowering these affective barriers is likely to increase service uptake and access, the sheer diversity of such barriers to service access described in this study suggests that there is no simple or homogenous solution that is universally effective.

Collectively, LGBTQ victim-survivors experience shared challenges in finding accessible, safe, and affirming services. These challenges assume distinctive manifestations, potentiate different harms, and reliably correspond to social variation and preexisting marginality. Individual victim-survivor's patterns of help-seeking behaviors and the affective impediments to service access are furthermore refracted through experiences of racism, economic disadvantage, and service gaps in nonurban localities. The range of experiences and challenges captured within our findings likewise demands a correspondingly nuanced approach to dismantling and/or lowering these affective barriers, but fundamentally affirms the need for concomitant access to a choice of safe services and referral options for people experiencing IPV, as well as a multifaceted approach to primary prevention, awareness, and advocacy.

Firstly, our findings affirm a need for more education and advocacy targeting LGBTQ people and service providers to encourage the recognition and successful identification of abusive dynamics and behaviors as IPV. These efforts should optimally aim to mold the public narrative around IPV to foster lay understandings that accommodate both nonphysical and nonheterogendered forms of IPV. Secondly, our findings demonstrate the need to comprehensively address the shame and embarrassment experienced by victim-survivors—both as a means of lessening the subjective distress associated with experiencing violence, as well as to improve service uptake among victim-survivors. As suggested by both participant reflections, as well as previous research (see Donovan & Hester, 2011), much of the shame affixed to victimhood can be located within broader cultural narratives that position victims as weak and feminized. This, in turn, can often be traced back to legacy discourses about IPV which centers on the figure of the “battered wife,” a beleaguered victim who lacks any appreciable agency in either her relationship or in the face of the violence wielded against her by here calcitrant spouse (Paradis et al., 2020).

For LGBTQ men, these gender-stereotypical understandings of intimate partner victimization often collide with masculine gender norms to compound feelings of shame and embarrassment or humiliation, and/or to obstruct the recognition of one's experiences as abuse. Concurrently, the denigrating associations between femininity and victimhood may result in LGBTQ women's disidentification with these narratives due to the stereotype threat posed. Regardless of gender, the dichotomous conceptualization of IPV along a victim/perpetrator binary further obfuscates the recognition of IPV, as well as the sources of support and safety available to victim-survivors (Donovan & Hester, 2011). Interventions aimed toward addressing the shame and stigma attached to IPV should therefore be grounded within an ethos of repair and community safety and should take precedence over punitive responses toward IPV (Lusby et al., 2022). Ideally, these interventions should be deployed in tandem with efforts to raise awareness and advocate for LGBTQ victim-survivors (Lusby et al., 2022).

Our findings further highlight community trust and goodwill as critical factors in service uptake and illustrate how negative experiences with professionalized service providers can often accumulate and circulate within LGBTQ communities. These negative opinions subsequently coalesce into powerful affective barriers to service uptake that often persist long after improvements to these services have been successfully undertaken. As such, community consultation and outreach must accompany the development of LGBTQ-specific service capabilities for these improvements to meaningfully contribute to the support options available to LGBTQ victim-survivors. Relatedly, both the uptake and accessibility of LGBTQ community-led services as well as mainstream services with LGBTQ-inclusive capabilities must be a cornerstone of service provision for LGBTQ victim-survivors. While both the uptake and accessibility of LGBTQ community-led services and mainstream services with LGBTQ-inclusive capabilities must be a cornerstone of service provision for LGBTQ victim-survivors, our findings illustrate the unique challenges that can arise within this context. This points to the need for a range of support options and referral pathways for LGBTQ victim-survivors, instead of siloing all service demand from this population into a small number of specialized services (Lusby et al., 2022).

### Limitations

Our chosen methodology places significant constraints on the generalizability of these findings. Likewise, as our sample was comprised predominantly of persons of Anglo-Celtic, and/or other white European ancestry, our findings may not adequately engage with the full range of racialized experiences with respect to this topic. As the limited evidence presented here suggests, however, these experiences are likely informed by experiences of racial minoritization and should be pursued by future research. Finally, because we utilized a convenience sample recruited through community networks, our participants likely represent a subset of the overall population of LGBTQ victim-survivors that are particularly well-embedded within these networks. Affective barriers relating to anticipated discrimination are likely to be understated within our findings, whereas those relating to entanglements with LGBTQ persons working in the FDSV sector may be more prominent.

## Conclusion

Like material, structural, and institutional barriers, affective barriers to service engagement among LGBTQ victim-survivors greatly constrain timely access to appropriate professionalized support. These barriers are closely intertwined with individual victim-survivors’ marginalized identities and experiences and are enmeshed with overarching structural and institutional inequality. Uniquely, affective barriers can perform a defensive or protective function, serving to limit or minimize the likelihood of a victim-survivor encountering discriminatory treatment during the help-seeking process. However, these barriers unfairly foreclose upon certain professionalized services as viable sources of support, irrespective of subsequent improvements to service inclusivity or LGBTQ-specific expertise—forming a critical barrier to service uptake and utilization. As such, service organizations attempting the diversification of their capabilities to encompass service provision to sexual and/or gender minorities are urged to consider complementing these improvements with community engagement, outreach, and other such efforts aimed toward lowering the affective barriers detailed herein.

## Notes

1. We use the acronym “LGBTQ” in this paper as the principal way of framing the scope of our enquiry, based upon the characteristics of our survey participants. In other places we use “LGBT” or “LGBTIQ” when referring to previous research conducted by others that was inclusive of different sections of the population of people who are diverse in their gender, sexuality, or sex characteristics.

2. *Brotherboy* is a label used by “Indigenous [Australian] transgender or gender diverse […] person who was assigned female at birth but inside they have a boy spirit, and they live through that boy spirit, and they take on male roles in […] society and […] community, and they live their lives as a male, and being a Brotherboy encompasses your gender identity as well as your cultural identity” (Gloria, 2014, as cited in Anae, 2020).
